# Impact of Quality Improvement Bundle on Neonatal Mortality in a District Hospital in Tanzania

**DOI:** 10.3390/children9071060

**Published:** 2022-07-15

**Authors:** Andrea Pietravalle, Luca Brasili, Francesco Cavallin, Margherita Piquè, Chiara Zavattero, Gaetano Azzimonti, Donald Micah Maziku, Dionis Erasto Leluko, Giovanni Putoto, Daniele Trevisanuto

**Affiliations:** 1 Doctors with Africa CUAMM, 35128 Padua, Italy; g.putoto@cuamm.org; 2 Doctors with Africa CUAMM, Dar es Salaam 23447, Tanzania; l.brasili@cuamm.org (L.B.); margherita.pique@gmail.com (M.P.); chiara.zavaretto@gmail.com (C.Z.); g.azzimonti@cuamm.org (G.A.); 3 Independent Statistician, 36020 Solagna, Italy; cescocava@libero.it; 4 St. John of the Cross, Tosamaganga Council Designated Hospital, Iringa 50201, Tanzania; dmaziku@yahoo.com (D.M.M.); dionisleluko97@gmail.com (D.E.L.); 5 Department of Woman’s and Child’s Health, University of Padua, 35122 Padua, Italy; daniele.trevisanuto@unipd.it

**Keywords:** neonatal mortality, low birth weight infants, quality improvement, B.A.B.I.E.S. Matrix tool

## Abstract

Background: The poor quality of care received by mothers and neonates in many limited-resource countries represents a main determinant of newborn mortality. Small and sick hospitalized newborns are the highest-risk population, and they should be one of the prime beneficiaries of quality-of-care interventions. This study aimed to evaluate the impact on neonatal mortality of quality improvement interventions which were implemented at Tosamaganga Council Designated Hospital, Iringa, Tanzania, between 2016 and 2020. Methods: A retrospective comparison between pre- and post-intervention periods was performed using the chi-square test and Fisher’s exact test. Effect sizes were reported as odds ratios with 95% confidence intervals. Results: The analysis included 5742 neonates admitted to the Special Care Unit (2952 in the pre-intervention period and 2790 in the post-intervention period). A decrease in mortality among infants with birth weight between 1500 and 2499 g (overall: odds ratio 0.49, 95% confidence interval 0.27–0.87; inborn: odds ratio 0.50, 95% confidence interval 0.27–0.93) was found. The analysis of cause-specific mortality showed a decrease in mortality for asphyxia (odds ratio 0.33, 95% confidence interval 0.12–0.87) among inborn infants with birth weight between 1500 and 2499 g. Conclusions: A quality improvement intervention was associated with decreased mortality among infants with birth weight between 1500 and 2499 g. Further efforts are needed to improve prognosis in very-low-birth-weight infants.

## 1. Introduction

Poor neonatal outcomes represent a significant global health burden [1], with a large proportion of neonatal deaths occurring in low-resource settings [2]. Over the years, under-five-year-old mortality (U5M) declined more significantly than neonatal mortality did, resulting in a larger contribution of neonatal deaths to the U5M rate [3]. The poor quality of care received by mothers and babies in many limited-resource countries contributes to the high levels of newborn mortality [4]. Quality improvement (QI) is defined as “better patient experience and outcomes achieved through changing provider behavior and organization through using a systematic change methods and strategies” [5] Recent evidence shows that quality improvement initiatives can reduce the burden of mortality and morbidity for hospitalized newborns in developing countries [6,7,8].

The aim of the present study was to evaluate the impact on neonatal mortality of quality improvement interventions implemented at Tosamaganga Council Designated Hospital, Iringa, Tanzania, between 2017 and 2020.

## 2. Materials and Methods

### 2.1. Study Design

This retrospective study compared “pre-intervention” (1 January–31 December 2016) and “post-intervention” (1 January–31 December 2020) mortality data of newborns admitted to the neonatal Special Care Unit (SCU) of Tosamaganga Hospital, Iringa, Tanzania. The periods were separated by a 3-year time span (2017–2019) which was needed to implement the quality improvement bundle.

### 2.2. Setting

Tosamaganga Hospital is a District Designated Hospital in the District of Iringa (Tanzania) and is the referral hospital for major obstetric emergencies for around 260,000 people living in the area. Every year, around 3000 deliveries and 500 admissions to the SCU occur at Tosamaganga Hospital. The SCU offers basic intensive care such as intravenous therapies, phototherapy and oxygen supplementation without non-invasive respiratory support and mechanical ventilation. Since January 2019, all babies discharged from the SCU are offered regular follow-up visits (to monitor clinical wellbeing, growth and neurological development) at the neonatal follow-up clinic during their first year of life.

### 2.3. Patients

All newborns admitted to the SCU of Tosamaganga Hospital (Iringa, Tanzania) during the study periods were included.

### 2.4. Interventions

During 2017–2019, a structured quality improvement process was implemented by Doctors with Africa CUAMM, an Italian nongovernmental organization operating in the field of healthcare in developing countries [9]. The interventions focused on improving infrastructure, equipment, training and use of clinical protocols, with a specific target on low-birth-weight infants (LBW, <2500 g) and pathologic newborns. Table 1 summarizes the area of interventions, timing and actions which were implemented during the process.

### 2.5. Outcome Measures

The main indicators were derived from the World Health Organization (WHO) B.A.B.I.E.S. Matrix tool (Birth weight group, Age at death, Boxes for an Intervention Evaluation System) [10], which is described in paragraph 2.7. The primary outcome measures included deaths/live births before discharge <1499 g (B.A.B.I.E.S. Matrix Cell 3), deaths/live births before discharge 1500–2499 g (B.A.B.I.E.S. Matrix Cell 7), and deaths/live births before discharge ≥2500 g (B.A.B.I.E.S. Matrix Cell 11). The secondary outcome measures included the mortality rates from asphyxia, infection and prematurity, according to the main causes of death (as defined by the tool for cells 3, 7 and 11).

### 2.6. Data Collection 

All data were retrospectively and anonymously collected from hospital charts by a researcher who was not involved in clinical activity. The researcher was not masked to the intervention period. Retrieved data did not contain any information that might be used to identify individual patients. 

### 2.7. Definitions

**Figure 1:**
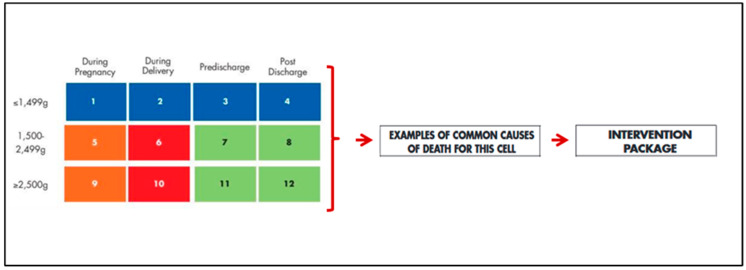
World Health Organization B.A.B.I.E.S. Matrix tool, modified from Joy Lawn et al. [10].

The B.A.B.I.E.S. Matrix tool works by segregating, organizing, analyzing and transforming data regarding fetal and neonatal deaths. Through stratification of data by weight and by moment of death, the tool guides identifications of the main problems related to pregnancy, labor, delivery and postnatal management, suggesting the causes and the appropriate interventions needed to reduce neonatal mortality (Figure 1). The stratification works on three birth weight categories (<1499, 1500–2499 and ≥2500 g) and on four time categories (during pregnancy: from 28 weeks of gestational age to the beginning of labor; during labor: from the beginning of labor to delivery time; pre-discharge: from delivery time to discharge time; post-discharge: from discharge to 28 days of life).

To identify the cause-specific mortality, the criteria reported by Mmbaga et al. [11] were used: (a) Birth asphyxia: birth asphyxia with weight >1000 g or gestational age >27 weeks; birth asphyxia and prematurity with gestational age ≥33 weeks and birth weight ≥2500 g or birth weight ≥1800 g if gestational age is unknown; neonatal encephalopathy with 5-min Apgar lower than 7; (b) Prematurity: prematurity; prematurity and asphyxia with gestational age <33 weeks and birth weight <2500 g or birth weight <1800 g if gestational age is unknown; respiratory distress syndrome in preterm; necrotizing enterocolitis; birth asphyxia gestational age <27 weeks or birth weight <1000 g; infection with gestational age <33 weeks; (c) Infection: neonatal infection; sepsis/septicemia; meningitis; pneumonia; impetigo neonatorum.

### 2.8. Statistical Analysis

Categorical data were summarized as frequencies and percentages. Comparisons between pre- and post-intervention periods were performed using the chi-square test and Fisher’s exact test. Effect sizes were reported as odds ratios with 95% confidence intervals for each outcome measure. All tests were 2-sided, and a *p*-value less than 0.05 was considered statistically significant. Adjustment for multiple testing was not performed due to the exploratory purpose of the study. Statistical analysis was performed using R 4.1 (R Foundation for Statistical Computing, Vienna, Austria) [12].

## 3. Results

The analysis included 2952 neonates admitted to the SCU in the pre-intervention period and 2790 neonates admitted to the SCU in the post-intervention period. The characteristics of deliveries and neonates are reported in Table 2. The baseline characteristics were clinically comparable between the two periods, with small changes in cesarean sections (36.4% vs. 30.7%, *p* < 0.0001), inborn neonates (97.9% vs. 96.6%, *p* < 0.0001) and LBW neonates (10.4% vs. 13.7%; *p* < 0.0001).

A comparison of neonatal mortality between pre- and post-intervention periods is summarized in Table 3. Overall mortality did not change after the implementation of the interventions (odds ratio 1.05, 95% confidence interval 0.78 to 1.41). However, there was a decrease in overall mortality among infants with birth weight between 1500 and 2499 g (odds ratio 0.49, 95% confidence interval 0.27–0.87) and in inborn neonates of the same birth weight category (odds ratio 0.50, 95% confidence interval 0.27–0.93). 

The analysis of cause-specific mortality found a decrease in mortality for asphyxia (odds ratio 0.33, 95% confidence interval 0.12–0.87) among infants with birth weight between 1500 and 2499 g and an increase in overall mortality for prematurity (odds ratio 1.82, 95% confidence interval 1.03–3.29). No statistically significant differences in mortality were observed among outborn neonates.

## 4. Discussion

Recent evidence suggests that an effective implementation of quality improvement in the care of small and sick newborns is possible in low-resource settings [6,7,8]. The literature indicates limitations in staff, equipment and protocols as the main barriers for such implementation and underlines the opportunity for meso-level and educational interventions [6]. This study reports neonatal mortality outcomes after a quality improvement intervention in a sub-Saharan setting. According to indications drawn from the literature, our quality improvement approach involved the use of meso and micro interventions (such as strengthening the facility’s infrastructure, continuous quality improvement, supervision, feedback, in-service training, distribution of referencing materials to providers, decision support and care coordination) and used mortality as the main outcome measure [6]. The decrease in mortality among infants with birth weight between 1500 and 2499 g supports the effectiveness of the implementation of specifically targeted interventions and indirectly underlines the importance of the B.A.B.I.E.S Matrix as a guiding tool for improving quality of neonatal care. The decrease in mortality for asphyxia among infants with birth weight between 1500 and 2499 g suggested an improvement in stabilization practices immediately after birth for low-birth-weight infants. Of note, we found increased overall mortality due to prematurity, which might likely be due to a bias in the definition of prematurity. As gestational age is rarely available in low-resource settings, a birth weight <1800 g was used to define prematurity [11]. Within this category, there was a larger number of babies with birth weight <1500 g in 2020 vs. that in 2016. Very-low-birth-weight newborns (<1500 g) are extremely fragile, and their mortality varies considerably among high-income (12–15%) and low–middle-income countries (21–43%) [13,14,15]. Unfortunately, reducing mortality in this subgroup of newborns requires massive human and economic resources, and the B.A.B.I.E.S Matrix tool suggests interventions on pre-pregnancy health and high-tech neonatal care that are very difficult to implement in low-resource settings.

The reader should be aware that reducing neonatal mortality may come at the cost of increased post-discharge morbidity in such vulnerable subjects. A follow-up service for high-risk newborns is currently active at the study site, but unfortunately, the high dropout rate makes any assessment difficult.

The present study has some limitations that should be considered by the reader. First, the retrospective design limited both the availability and quality of data. Second, the quality improvement was implemented in a sub-Saharan referral hospital, hence the generalizability of the findings should be limited to similar settings. Third, adjustment for multiple testing was not performed due to the exploratory purpose of the study, hence we suggest caution in the interpretation of the results. Finally, we could not discriminate the specific impact of each component of the bundle.

Future interventions for reducing morbidity and mortality in this setting would focus on applying new strategies such as the use of devices for non-invasive respiratory support and improving good practices of infection prevention, nutritional support and maintenance of normothermia. Hypothermia, hypoglycemia and infections are the main causes of death in the neonatal period, and their prevention is even more important in very-low-birth-weight newborns. To this end, it would be necessary to pay even greater attention to the management of vascular access, parenteral fluids and enteral nutrition. Strengthening Kangaroo mother care and close monitoring of temperature and blood sugar would also be essential.

## 5. Conclusions

A quality improvement process based on meso and micro interventions was associated with decreased mortality among infants with birth weight between 1500 and 2499 g. Further efforts are needed to improve prognosis in very-low-birth-weight infants.

## Figures and Tables

**Table 1:** Summary of the quality improvement process which was implemented in 2017–2019 at Tosamaganga Hospital (Iringa, Tanzania).

Area of Intervention	Year	Action
Infrastructures	January 2017	A Neonatal ward was constructed near the Maternity Ward, divided into three areas: Neonatal Intensive Care Unit (one room), Neonatal Sub-intensive Care Unit (one room) and Kangaroo Mother Care Unit (two rooms)
Equipment	January 2017	Four oxygen concentrators (increased over the years up to 10), two phototherapy machines, four infusion pumps and a syringe pump, a capillary hemoglobin dosing machine and an electric aspirator were purchased. The staff received training on their use.
Protocols	2017 and 2019	Operational protocols were updated and presented to the staff in dedicated training sessions. Laminated copies of the most commonly used protocols were displayed for quick consultation even by on-call staff during night shifts and holidays. A further update of the ward guidelines was carried out in 2019, in light of the publication of the first edition of the national neonatal guidelines.
Procedures	January 2017	New procedures were introduced: antenatal administration of dexamethasone for lung maturity and magnesium sulfate for neuroprotection, positioning of an umbilical venous catheter in newborns weighing <1200 g, administration of paracetamol in newborns with suspected patent ductus arteriosus, administration of hydrocortisone in newborns with oxygen dependence and suspected bronchopulmonary dysplasia.
Staff	2017	A dedicated nursing team was created, consisting of 5 nurses (increased over the years up to 8). From February 2017, a Tanzanian doctor started working in Neonatology.
Training activity	2017–2019	Over years, the Neonatal Unit and Maternity Ward staff were periodically trained on partogram use and interpretation, management of a complicated pregnancy (gestational hypertension, gestational diabetes, prolonged rupture of the membranes); management of labor and delivery (1st, 2nd, 3rd stage), prolonged rupture of the membranes, complicated labor and the most common maternal peripartum complications, neonatal resuscitation, management of common neonatal severe conditions (sepsis, jaundice, asphyxia, prematurity, respiratory distress syndrome), essential newborn care and care of low-birth-weight and very-low-birth-weight infants.

**Table 2:** Baseline characteristics of neonates admitted to the SCU in the pre- vs. post-intervention periods.

	Pre-Intervention	Post-Intervention Period:	*p*-Value
	Period:		
**Deliveries**	N = 2901	N = 2732	-
**Mode of delivery:**			<0.0001
Caesarean section	1056/2901 (36.4%)	840/2732 (30.7%)	
Vaginal delivery	1845/2901 (63.6%)	1892/2732 (69.3%)	
**Twin deliveries**	50/2901 (1.7%)	55/2732 (2.0%)	0.48
**Neonates**	N = 2952	N = 2790	
**Inborn neonates**	2890/2952 (97.9%)	2675/2790 (95.6%)	<0.0001
**Males**	1472/2952 (49.9%)	1370/2790 (49.1%)	0.58
**Birth weight:**			<0.0001
≤1499 g	24/2952 (0.8%)	63/2790 (2.3%)	
1500–2499 g	282/2952 (9.6%)	318/2790 (11.4%)	
≥2500 g	2646/2952 (89.6%)	2409/2790 (86.3%)	
**5-min Apgar score < 7 (only inborn)**	121/2890 (4.2%)	109/2675 (4.1%)	0.89

Data were summarized as n/N (%).

**Table 3:** Comparison of mortality of neonates admitted to the SCU in the pre- vs. post-intervention periods.

Outcome Measure	Pre-Intervention Period:	Post-Intervention Period:	Post vs. Pre Comparison: Odds Ratio (95% Confidence Interval)	*p*-Value
All neonates	N = 2952	N = 2790	*-*	*-*
Overall mortality	87/2952 (2.9%)	92/2790 (3.3%)	1.05 (0.78 to 1.41)	0.49
Mortality in BW categories:				
≤1499 g	13/24 (54.2%)	31/63 (49.2%)	0.81 (0.32 to 2.10)	0.86
1500–2499 g	34/282 (12.1%)	20/318 (6.3%)	0.49 (0.27 to 0.87)	0.02
≥2500 g	40/2646 (1.5%)	41/2409 (1.7%)	1.12 (0.72 to 1.75)	0.67
Mortality for prematurity	21/2952 (0.7%)	36/2790 (1.3%)	1.82 (1.03 to 3.29)	0.04
Mortality for asphyxia:				
Overall	44/2952 (1.5%)	40/2790 (1.4%)	0.96 (0.62 to 1.47)	0.94
≤1499 g	0/24 (0.0%)	0/63 (0.0%)	NA	NA
1500–2499 g	15/282 (5.3%)	7/318 (2.2%)	0.40 (0.16 to 1.00)	0.07
≥2500 g	29/2646 (1.1%)	33/2409 (1.4%)	1.25 (0.75 to 2.07)	0.45
Mortality for infection:				
Overall	12/2952 (0.4%)	6/2790 (0.2%)	0.52 (0.19 to 1.40)	0.29
≤1499 g	0/24 (0.0%)	0/63 (0.0%)	NA	NA
1500–2499 g	5/282 (1.8%)	2/318 (0.6%)	0.35 (0.06 to 1.82)	0.26
≥2500 g	7/2646 (0.3%)	4/2409 (0.2%)	0.62 (0.18 to 2.14)	0.55
**Inborn neonates**	**N = 2890**	**N = 2675**	***-***	***-***
Overall mortality	77/2890 (2.7%)	73/2675 (2.7%)	1.02 (0.74 to 1.41)	0.94
Mortality in BW categories:				
≤1499 g	10/15 (66.7%)	22/30 (73.3%)	1.37 (0.35 to 5.27)	0.90
1500–2499 g	29/249 (11.6%)	18/287 (6.3%)	0.50 (0.27 to 0.93)	0.04
≥2500 g	38/2626 (1.4%)	33/2358 (1.4%)	0.96 (0.60 to 1.54)	0.98
Mortality for prematurity	15/2890 (0.5%)	26/2675 (1.0%)	1.88 (0.99 to 3.55)	0.07
Mortality for asphyxia:				
Overall	43/2890 (1.5%)	33/2675 (1.2%)	0.82 (0.52 to 1.30)	0.48
≤1499 g	0/15 (0.0%)	0/30 (0.0%)	NA	NA
1500–2499 g	15/249 (6.0%)	6/287 (2.1%)	0.33 (0.12 to 0.87)	0.03
≥2500 g	28/2626 (1.1%)	27/2358 (1.1%)	1.07 (0.63 to 1.82)	0.90
Mortality for infection:				
Overall	10/2890 (0.3%)	5/2675 (0.2%)	0.53 (0.18 to 1.57)	0.38
≤1499 g	0/15 (0.0%)	0/30 (0.0%)	NA	NA
1500–2499 g	4/269 (1.5%)	2/287 (0.7%)	0.46 (0.08 to 2.55)	0.44
≥2500 g	6/2626 (0.2%)	3/2358 (0.1%)	0.55 (0.13 to 2.22)	0.51
**Outborn neonates**	**N = 62**	**N = 115**	***-***	***-***
Overall mortality	10/62 (16.1%)	19/115 (16.5%)	1.02 (0.44 to 2.37)	0.99
Mortality in BW categories:				
≤1499 g	3/9 (33.3%)	9/33 (27.3%)	0.75 (0.15 to 3.65)	0.69
1500–2499 g	5/33 (15.2%)	2/31 (6.5%)	0.38 (0.06 to 2.15)	0.42
≥2500 g	2/20 (10.0%)	8/51 (15.7%)	1.67 (0.32 to 8.66)	0.71
Mortality for prematurity	6/62 (9.7%)	10/115 (8.7%)	0.88 (0.30 to 1.57)	0.99
Mortality for asphyxia:				
Overall	1/62 (1.6%)	7/115 (6.1%)	3.95 (0.47 to 32.89)	0.26
≤1499 g	0/9 (0.0%)	0/33 (0.0%)	NA	NA
1500–2499 g	0/33 (0.0%)	1/31 (3.2%)	3.29 (0.12 to 89.97)	0.48
≥2500 g	1/20 (5.0%)	6/51 (11.8%)	2.53 (0.28 to 22.49)	0.66
Mortality for infection:				
Overall	2/62 (3.2%)	1/115 (0.9%)	0.26 (0.02 to 2.96)	0.28
≤1499 g	0/9 (0.0%)	0/33 (0.0%)	NA	NA
1500–2499 g	1/33 (3.0%)	0/31 (0.0%)	0.34 (0.01 to 8.76)	0.99
≥2500 g	1/20 (5.0%)	1/51 (2.0%)	0.38 (0.02 to 6.38)	0.48

Data were summarized as n/N (%).

## Data Availability

The datasets used and/or analyzed during the current study are available from the corresponding author on reasonable request.
